# Bis(benzimidazolium) naphthalene-1,5-disulfonate trihydrate

**DOI:** 10.1107/S1600536808005916

**Published:** 2008-03-05

**Authors:** Zi-Liang Wang, Lin-Yu Jin, Lin-Heng Wei

**Affiliations:** a Institute of Molecular and Crystal Engineering, College of Chemistry and Chemical Engineering, Henan University, Kaifeng 475001, People’s Republic of China; b College of Chemistry and Chemical Engineering, Henan University, Kaifeng 475001, People’s Republic of China; c College of the Environment and Planning, Henan University, Kaifeng 475001, People’s Republic of China

## Abstract

The title compound, 2C_7_H_7_N_2_^+^·C_10_H_6_O_6_S_2_^2−^·3H_2_O, consists of two crystallographically independent benzimidazolium cations, two independent naphthalene-1,5-disulfonate dianions (both generated by inversion) and three water mol­ecules. These components construct an infinite three-dimensional framework in the crystal structure *via* O—H⋯O and N—H⋯O hydrogen bonds.

## Related literature

For related literature, see: Wang & Wei (2007 ▶).
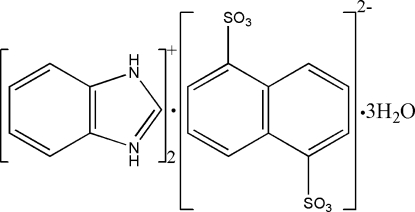


## Experimental

### 

#### Crystal data

2C_7_H_7_N_2_^+^·C_10_H_6_O_6_S_2_^2−^·3H_2_O
*M* *_r_* = 578.61
Triclinic, 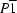

*a* = 8.372 (4) Å
*b* = 9.889 (5) Å
*c* = 17.044 (8) Å
α = 80.914 (8)°
β = 87.557 (9)°
γ = 73.641 (8)°
*V* = 1337.0 (11) Å^3^
*Z* = 2
Mo *K*α radiation
μ = 0.26 mm^−1^
*T* = 296 (2) K
0.15 × 0.12 × 0.04 mm

#### Data collection

Bruker SMART APEX CCD diffractometer
Absorption correction: multi-scan (*SADABS*; Bruker, 2001 ▶) *T*_min_ = 0.962, *T*_max_ = 0.990
6891 measured reflections
4670 independent reflections
3494 reflections with *I* > 2σ(*I*)
*R*_int_ = 0.020

#### Refinement

*R*[*F* ^2^ > 2σ(*F* ^2^)] = 0.043
*wR*(*F* ^2^) = 0.110
*S* = 1.06
4670 reflections
392 parameters
37 restraints
H atoms treated by a mixture of independent and constrained refinement
Δρ_max_ = 0.30 e Å^−3^
Δρ_min_ = −0.33 e Å^−3^

### 

Data collection: *SMART* (Bruker, 2001 ▶); cell refinement: *SAINT-Plus* (Bruker, 2001 ▶); data reduction: *SAINT-Plus*; program(s) used to solve structure: *SHELXS97* (Sheldrick, 2008 ▶); program(s) used to refine structure: *SHELXL97* (Sheldrick, 2008 ▶); molecular graphics: *PLATON* (Spek, 2003 ▶); software used to prepare material for publication: *PLATON*.

## Supplementary Material

Crystal structure: contains datablocks global, I. DOI: 10.1107/S1600536808005916/hb2700sup1.cif

Structure factors: contains datablocks I. DOI: 10.1107/S1600536808005916/hb2700Isup2.hkl

Additional supplementary materials:  crystallographic information; 3D view; checkCIF report

## Figures and Tables

**Table 1:** Hydrogen-bond geometry (Å, °)

*D*—H⋯*A*	*D*—H	H⋯*A*	*D*⋯*A*	*D*—H⋯*A*
N1—H1*A*⋯O7^i^	0.898 (10)	1.820 (12)	2.708 (3)	170 (3)
N2—H2*A*⋯O4^ii^	0.887 (18)	1.885 (19)	2.762 (3)	170 (3)
N3—H3*A*⋯O3^iii^	0.895 (10)	1.937 (12)	2.812 (3)	166 (2)
N4—H4*A*⋯O8^iv^	0.899 (10)	1.883 (11)	2.771 (3)	169 (2)
O7—H7*B*⋯O5^v^	0.852 (10)	2.169 (19)	2.839 (3)	135 (2)
O8—H8*A*⋯O2^vi^	0.844 (10)	1.982 (10)	2.826 (3)	178 (3)
O7—H7*A*⋯O1	0.853 (10)	1.999 (12)	2.823 (3)	162 (3)
O8—H8*B*⋯O6	0.847 (10)	1.963 (10)	2.809 (3)	176 (3)
O9—H9*B*⋯O1^iv^	0.863 (10)	2.157 (18)	2.970 (3)	157 (3)

Symmetry codes: (i) 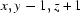; (ii) 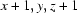; (iii) 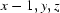; (iv) 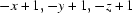; (v) 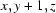; (vi) 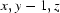.

**Fig. 1.**
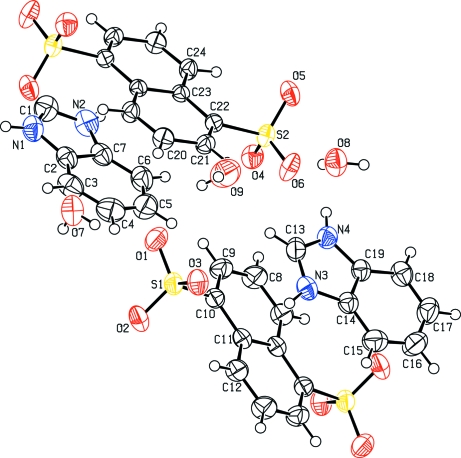
The molecular structure of (I). Displacement ellipsoids for non-H atoms are drawn at the 30% probability level. Hydrogen bonds are shown as dashed lines. Unlabeled atoms in the C8 anion are related to labeled atoms by (1 - x, 1 - y, 1 - z). Unlabeled atoms in the C20 anion are related to labeled atoms by (1 - x, 1 - y, -z).

**Fig. 2.**
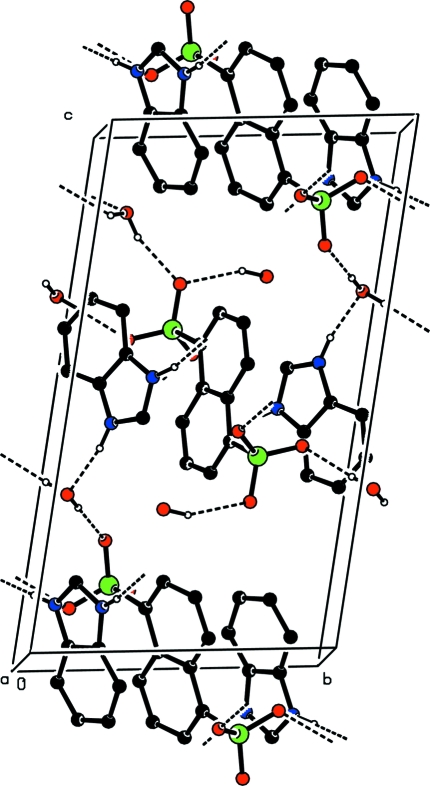
The crystal packing of (I). Hydrogen bonds are shown as dashed lines. For clarity, H atoms not involved in hydrogen bonds are omitted.

## supplementary crystallographic information

### Comment

This work continues our previous synthetic and structural studies of supramolecular interactions in aromatic molecular salts and adducts (Wang & Wei, 2007). Herein we report the structure of the title salt, (I).

The title compound (I) contains two independent benzimidazolium cations, two naphthalene-1,5-disulfonate dianions and three water molecules (Fig. 1). Each of the dianions occupies a special position on an inversion centre. Therefore, the asymmetric unit of the crystal structure is composed of two half naphthalene-1,5-disulfonate dianions, two benzimidazolium cations and three water molecules.

These ions and molecules are finally organized into an infinite three-dimensional framework through N—H···O and O—H···O hydrogen bonds (Fig. 2 and Table 1).

### Experimental

A 5-ml ethanol solution of benzimidazole (1.00 mmol, 0.118 g) was added to an aqueous solution (25 ml) of naphthalene-1,5-disulfonic acid (0.50 mmol, 0.15 g). The mixture was stirred for 10 minutes at 373 K. The solution was filtered, and the filtrate was allowed to stand at room temperature. After several days, colourless blocks of (I) were recovered.

### Refinement

The H atoms bonded to N and O were located in a difference map and refined with distance restraints [N—H = 0.90 (1) Å, water O—H = 0.85 (1)Å and H···H = 1.34 (1) Å]; their *U*_iso_ values were freely refined.

The C-bound H atoms were positioned geometrically (C—H = 0.93 Å) and refined as riding with *U*_iso_(H) = 1.2*U*_eq_(C).

### Crystal data

**Table:**

2C_7_H_7_N_2_^+^·C_10_H_6_O_6_S_2_^2–^·3H_2_O	*Z* = 2
*M**_r_* = 578.61	*F*_000_ = 604
Triclinic, *P*1	*D*_x_ = 1.437 Mg m^−^^3^
Hall symbol: -P 1	Mo *K*α radiation λ = 0.71073 Å
*a* = 8.372 (4) Å	Cell parameters from 3010 reflections
*b* = 9.889 (5) Å	θ = 2.5–28.2º
*c* = 17.044 (8) Å	µ = 0.26 mm^−^^1^
α = 80.914 (8)º	*T* = 296 (2) K
β = 87.557 (9)º	Block, colourless
γ = 73.641 (8)º	0.15 × 0.12 × 0.04 mm
*V* = 1337.0 (11) Å^3^	

### Data collection

**Table:**

Bruker SMART APEX CCD diffractometer	4670 independent reflections
Radiation source: fine-focus sealed tube	3494 reflections with *I* > 2σ(*I*)
Monochromator: graphite	*R*_int_ = 0.020
*T* = 296(2) K	θ_max_ = 25.0º
ω scans	θ_min_ = 2.2º
Absorption correction: multi-scan(SADABS; Bruker, 2001)	*h* = −9→6
*T*_min_ = 0.962, *T*_max_ = 0.990	*k* = −11→10
6891 measured reflections	*l* = −20→20

### Refinement

**Table:**

Refinement on *F*^2^	Secondary atom site location: difference Fourier map
Least-squares matrix: full	Hydrogen site location: difmap and geom
*R*[*F*^2^ > 2σ(*F*^2^)] = 0.043	H atoms treated by a mixture of independent and constrained refinement
*wR*(*F*^2^) = 0.110	*w* = 1/[σ^2^(*F*_o_^2^) + (0.0559*P*)^2^ + 0.0198*P*] where *P* = (*F*_o_^2^ + 2*F*_c_^2^)/3
*S* = 1.06	(Δ/σ)_max_ < 0.001
4670 reflections	Δρ_max_ = 0.30 e Å^−^^3^
392 parameters	Δρ_min_ = −0.33 e Å^−^^3^
37 restraints	Extinction correction: none
Primary atom site location: structure-invariant direct methods	

### Special details

**Table:**

Geometry. All e.s.d.'s (except the e.s.d. in the dihedral angle between two l.s. planes) are estimated using the full covariance matrix. The cell e.s.d.'s are taken into account individually in the estimation of e.s.d.'s in distances, angles and torsion angles; correlations between e.s.d.'s in cell parameters are only used when they are defined by crystal symmetry. An approximate (isotropic) treatment of cell e.s.d.'s is used for estimating e.s.d.'s involving l.s. planes.
Refinement. Refinement of *F*^2^ against ALL reflections. The weighted *R*-factor *wR* and goodness of fit *S* are based on *F*^2^, conventional *R*-factors *R* are based on *F*, with *F* set to zero for negative *F*^2^. The threshold expression of *F*^2^ > σ(*F*^2^) is used only for calculating *R*-factors(gt) *etc*. and is not relevant to the choice of reflections for refinement. *R*-factors based on *F*^2^ are statistically about twice as large as those based on *F*, and *R*- factors based on ALL data will be even larger.

### Fractional atomic coordinates and isotropic or equivalent isotropic displacement parameters (Å^2^)

**Table:**

	*x*	*y*	*z*	*U*_iso_*/*U*_eq_	
S1	0.75176 (8)	0.65881 (6)	0.37087 (4)	0.04129 (19)	
S2	0.39100 (7)	0.25493 (6)	0.14588 (3)	0.03710 (17)	
N1	0.8368 (3)	0.0653 (2)	1.09595 (14)	0.0502 (6)	
H1A	0.794 (4)	−0.007 (2)	1.1155 (19)	0.098 (12)*	
N2	0.9885 (3)	0.2131 (2)	1.08427 (15)	0.0505 (6)	
H2A	1.065 (3)	0.253 (3)	1.0965 (18)	0.082 (11)*	
N3	0.0749 (3)	0.7339 (2)	0.48285 (14)	0.0460 (5)	
H3A	0.032 (3)	0.670 (2)	0.4660 (15)	0.063 (9)*	
N4	0.1689 (3)	0.8418 (2)	0.56460 (12)	0.0432 (5)	
H4A	0.192 (3)	0.865 (3)	0.6108 (9)	0.056 (8)*	
O1	0.7659 (2)	0.65613 (18)	0.28505 (10)	0.0554 (5)	
O2	0.6945 (2)	0.80262 (17)	0.39064 (11)	0.0571 (5)	
O3	0.9056 (2)	0.57458 (17)	0.41288 (10)	0.0473 (4)	
O4	0.21223 (19)	0.33257 (17)	0.14166 (10)	0.0457 (4)	
O5	0.4281 (2)	0.13448 (16)	0.10230 (10)	0.0490 (5)	
O6	0.4518 (2)	0.21705 (18)	0.22760 (10)	0.0545 (5)	
O7	0.6702 (3)	0.8721 (2)	0.15255 (12)	0.0636 (6)	
H7A	0.695 (3)	0.822 (3)	0.1982 (9)	0.072 (5)*	
H7B	0.575 (2)	0.928 (3)	0.1601 (16)	0.096 (14)*	
O8	0.7493 (3)	0.0547 (2)	0.30528 (12)	0.0573 (5)	
H8A	0.733 (3)	−0.0213 (17)	0.3299 (15)	0.067 (5)*	
H8B	0.657 (2)	0.102 (3)	0.283 (2)	0.130 (17)*	
O9	0.0253 (4)	0.6345 (3)	0.73328 (16)	0.0971 (8)	
H9A	−0.066 (2)	0.618 (3)	0.719 (2)	0.081 (5)*	
H9B	0.089 (3)	0.5484 (16)	0.743 (2)	0.101 (6)*	
C1	0.9359 (3)	0.1177 (3)	1.13375 (17)	0.0546 (7)	
H1	0.9641	0.0912	1.1873	0.065*	
C2	0.8228 (3)	0.1305 (2)	1.01695 (16)	0.0421 (6)	
C3	0.7326 (3)	0.1173 (3)	0.95341 (18)	0.0548 (7)	
H3	0.6675	0.0541	0.9585	0.066*	
C4	0.7446 (4)	0.2034 (3)	0.88195 (18)	0.0619 (8)	
H4	0.6861	0.1975	0.8380	0.074*	
C5	0.8424 (4)	0.2990 (3)	0.87418 (18)	0.0626 (8)	
H5	0.8468	0.3547	0.8252	0.075*	
C6	0.9320 (3)	0.3128 (3)	0.93678 (17)	0.0532 (7)	
H6	0.9967	0.3763	0.9314	0.064*	
C7	0.9205 (3)	0.2262 (2)	1.00908 (16)	0.0428 (6)	
C8	0.4007 (3)	0.4475 (3)	0.37232 (15)	0.0514 (7)	
H8	0.3477	0.4186	0.3338	0.062*	
C9	0.5171 (3)	0.5243 (3)	0.34934 (15)	0.0455 (6)	
H9	0.5410	0.5452	0.2957	0.055*	
C10	0.5963 (3)	0.5689 (2)	0.40503 (13)	0.0360 (5)	
C11	0.5595 (3)	0.5398 (2)	0.48859 (13)	0.0348 (5)	
C12	0.6357 (3)	0.5851 (3)	0.54888 (14)	0.0442 (6)	
H12	0.7114	0.6378	0.5346	0.053*	
C13	0.0892 (3)	0.7450 (3)	0.55844 (16)	0.0478 (7)	
H13	0.0493	0.6927	0.6011	0.057*	
C14	0.1487 (3)	0.8290 (2)	0.43592 (14)	0.0392 (6)	
C15	0.1657 (3)	0.8615 (3)	0.35337 (16)	0.0541 (7)	
H15	0.1264	0.8154	0.3181	0.065*	
C16	0.2444 (4)	0.9660 (3)	0.32755 (17)	0.0620 (8)	
H16	0.2584	0.9907	0.2733	0.074*	
C17	0.3036 (3)	1.0360 (3)	0.38033 (18)	0.0578 (8)	
H17	0.3552	1.1060	0.3601	0.069*	
C18	0.2877 (3)	1.0043 (3)	0.46113 (16)	0.0477 (6)	
H18	0.3274	1.0507	0.4960	0.057*	
C19	0.2086 (3)	0.8982 (2)	0.48839 (14)	0.0369 (6)	
C20	0.6767 (3)	0.5287 (2)	0.11047 (14)	0.0394 (6)	
H20	0.7486	0.5548	0.1414	0.047*	
C21	0.6018 (3)	0.4208 (2)	0.14309 (13)	0.0357 (5)	
H21	0.6249	0.3764	0.1953	0.043*	
C22	0.4946 (3)	0.3809 (2)	0.09785 (13)	0.0308 (5)	
C23	0.4614 (3)	0.4453 (2)	0.01605 (12)	0.0288 (5)	
C24	0.3560 (3)	0.4050 (2)	−0.03388 (13)	0.0353 (5)	
H24	0.3084	0.3323	−0.0139	0.042*	

### Atomic displacement parameters (Å^2^)

**Table:**

	*U*^11^	*U*^22^	*U*^33^	*U*^12^	*U*^13^	*U*^23^
S1	0.0484 (4)	0.0338 (3)	0.0428 (4)	−0.0151 (3)	−0.0003 (3)	−0.0022 (3)
S2	0.0385 (4)	0.0360 (3)	0.0376 (3)	−0.0162 (3)	0.0002 (3)	0.0027 (3)
N1	0.0443 (13)	0.0410 (13)	0.0639 (16)	−0.0139 (11)	0.0030 (11)	−0.0011 (11)
N2	0.0383 (13)	0.0498 (14)	0.0660 (16)	−0.0161 (11)	−0.0060 (11)	−0.0082 (12)
N3	0.0469 (13)	0.0372 (12)	0.0571 (15)	−0.0157 (10)	−0.0011 (11)	−0.0087 (11)
N4	0.0488 (13)	0.0396 (12)	0.0397 (13)	−0.0117 (10)	−0.0042 (10)	−0.0018 (10)
O1	0.0689 (13)	0.0589 (12)	0.0399 (10)	−0.0246 (10)	0.0042 (9)	−0.0003 (9)
O2	0.0714 (13)	0.0312 (9)	0.0693 (13)	−0.0159 (9)	0.0042 (10)	−0.0072 (9)
O3	0.0444 (10)	0.0422 (10)	0.0567 (11)	−0.0170 (8)	−0.0044 (8)	−0.0020 (8)
O4	0.0346 (10)	0.0478 (10)	0.0541 (11)	−0.0140 (8)	0.0058 (8)	−0.0033 (8)
O5	0.0555 (11)	0.0317 (9)	0.0634 (12)	−0.0180 (8)	0.0009 (9)	−0.0069 (8)
O6	0.0642 (12)	0.0609 (12)	0.0395 (10)	−0.0306 (10)	−0.0067 (9)	0.0153 (8)
O7	0.0655 (15)	0.0497 (13)	0.0740 (15)	−0.0202 (12)	−0.0078 (11)	0.0045 (10)
O8	0.0676 (14)	0.0493 (12)	0.0582 (13)	−0.0246 (11)	−0.0130 (11)	0.0009 (10)
O9	0.109 (2)	0.0876 (17)	0.0720 (16)	0.0001 (16)	0.0210 (16)	−0.0007 (14)
C1	0.0467 (17)	0.0544 (17)	0.0579 (18)	−0.0094 (14)	−0.0031 (14)	−0.0022 (14)
C2	0.0341 (14)	0.0358 (14)	0.0562 (17)	−0.0083 (11)	0.0035 (12)	−0.0099 (12)
C3	0.0445 (16)	0.0523 (17)	0.074 (2)	−0.0178 (13)	0.0072 (14)	−0.0234 (15)
C4	0.0578 (19)	0.069 (2)	0.061 (2)	−0.0145 (16)	−0.0041 (15)	−0.0214 (16)
C5	0.065 (2)	0.0580 (19)	0.0579 (19)	−0.0107 (16)	0.0059 (16)	−0.0032 (15)
C6	0.0507 (17)	0.0434 (15)	0.0679 (19)	−0.0184 (13)	0.0076 (15)	−0.0078 (14)
C7	0.0331 (14)	0.0369 (14)	0.0585 (17)	−0.0084 (11)	0.0032 (12)	−0.0112 (12)
C8	0.0587 (17)	0.0617 (17)	0.0434 (16)	−0.0275 (15)	−0.0076 (13)	−0.0147 (13)
C9	0.0541 (16)	0.0511 (15)	0.0341 (13)	−0.0175 (13)	−0.0024 (12)	−0.0087 (12)
C10	0.0384 (14)	0.0325 (12)	0.0363 (13)	−0.0083 (10)	−0.0013 (11)	−0.0054 (10)
C11	0.0360 (13)	0.0296 (12)	0.0400 (13)	−0.0095 (10)	−0.0027 (10)	−0.0074 (10)
C12	0.0474 (15)	0.0487 (15)	0.0441 (15)	−0.0229 (13)	−0.0004 (12)	−0.0118 (12)
C13	0.0498 (16)	0.0364 (14)	0.0532 (17)	−0.0104 (12)	0.0041 (13)	0.0014 (12)
C14	0.0350 (13)	0.0358 (13)	0.0450 (15)	−0.0080 (11)	0.0009 (11)	−0.0047 (11)
C15	0.0566 (18)	0.0572 (17)	0.0471 (16)	−0.0108 (14)	−0.0004 (13)	−0.0130 (13)
C16	0.067 (2)	0.068 (2)	0.0452 (17)	−0.0154 (17)	0.0105 (15)	−0.0002 (15)
C17	0.0546 (18)	0.0537 (17)	0.0628 (19)	−0.0193 (15)	0.0120 (15)	0.0027 (15)
C18	0.0410 (15)	0.0443 (15)	0.0606 (18)	−0.0165 (12)	0.0006 (13)	−0.0074 (13)
C19	0.0326 (13)	0.0336 (13)	0.0413 (14)	−0.0058 (11)	−0.0028 (11)	−0.0020 (11)
C20	0.0410 (14)	0.0432 (14)	0.0399 (14)	−0.0193 (12)	−0.0071 (11)	−0.0076 (11)
C21	0.0407 (14)	0.0372 (13)	0.0294 (12)	−0.0121 (11)	−0.0022 (10)	−0.0030 (10)
C22	0.0314 (12)	0.0278 (12)	0.0329 (12)	−0.0079 (10)	0.0016 (10)	−0.0045 (9)
C23	0.0276 (12)	0.0273 (12)	0.0319 (12)	−0.0078 (9)	0.0037 (10)	−0.0062 (10)
C24	0.0381 (13)	0.0343 (13)	0.0379 (13)	−0.0185 (11)	−0.0001 (10)	−0.0030 (10)

### Geometric parameters (Å, °)

**Table:**

S1—O2	1.4558 (18)	C5—H5	0.9300
S1—O1	1.4663 (19)	C6—C7	1.402 (4)
S1—O3	1.4665 (18)	C6—H6	0.9300
S1—C10	1.802 (2)	C8—C12^i^	1.373 (3)
S2—O5	1.4555 (18)	C8—C9	1.404 (3)
S2—O6	1.4586 (18)	C8—H8	0.9300
S2—O4	1.4761 (18)	C9—C10	1.373 (3)
S2—C22	1.789 (2)	C9—H9	0.9300
N1—C1	1.329 (3)	C10—C11	1.445 (3)
N1—C2	1.393 (3)	C11—C12	1.423 (3)
N1—H1A	0.898 (10)	C11—C11^i^	1.442 (4)
N2—C1	1.322 (3)	C12—C8^i^	1.373 (3)
N2—C7	1.397 (3)	C12—H12	0.9300
N2—H2A	0.887 (18)	C13—H13	0.9300
N3—C13	1.323 (3)	C14—C19	1.394 (3)
N3—C14	1.398 (3)	C14—C15	1.403 (3)
N3—H3A	0.895 (10)	C15—C16	1.382 (4)
N4—C13	1.330 (3)	C15—H15	0.9300
N4—C19	1.396 (3)	C16—C17	1.401 (4)
N4—H4A	0.899 (10)	C16—H16	0.9300
O7—H7A	0.853 (10)	C17—C18	1.373 (4)
O7—H7B	0.852 (10)	C17—H17	0.9300
O8—H8A	0.844 (10)	C18—C19	1.402 (3)
O8—H8B	0.847 (10)	C18—H18	0.9300
O9—H9A	0.871 (10)	C20—C24^ii^	1.367 (3)
O9—H9B	0.863 (10)	C20—C21	1.414 (3)
C1—H1	0.9300	C20—H20	0.9300
C2—C3	1.389 (4)	C21—C22	1.382 (3)
C2—C7	1.403 (3)	C21—H21	0.9300
C3—C4	1.389 (4)	C22—C23	1.442 (3)
C3—H3	0.9300	C23—C24	1.425 (3)
C4—C5	1.403 (4)	C23—C23^ii^	1.436 (4)
C4—H4	0.9300	C24—C20^ii^	1.367 (3)
C5—C6	1.375 (4)	C24—H24	0.9300
			
O2—S1—O1	113.02 (11)	C9—C8—H8	119.7
O2—S1—O3	112.36 (11)	C10—C9—C8	120.8 (2)
O1—S1—O3	111.76 (11)	C10—C9—H9	119.6
O2—S1—C10	107.58 (11)	C8—C9—H9	119.6
O1—S1—C10	105.71 (11)	C9—C10—C11	120.7 (2)
O3—S1—C10	105.83 (10)	C9—C10—S1	117.92 (18)
O5—S2—O6	113.58 (11)	C11—C10—S1	121.33 (17)
O5—S2—O4	111.49 (10)	C12—C11—C11^i^	118.8 (3)
O6—S2—O4	111.78 (11)	C12—C11—C10	123.3 (2)
O5—S2—C22	108.32 (10)	C11^i^—C11—C10	117.9 (3)
O6—S2—C22	106.06 (10)	C8^i^—C12—C11	121.2 (2)
O4—S2—C22	105.02 (10)	C8^i^—C12—H12	119.4
C1—N1—C2	108.7 (2)	C11—C12—H12	119.4
C1—N1—H1A	127 (2)	N3—C13—N4	110.2 (2)
C2—N1—H1A	124 (2)	N3—C13—H13	124.9
C1—N2—C7	109.1 (2)	N4—C13—H13	124.9
C1—N2—H2A	124 (2)	C19—C14—N3	106.2 (2)
C7—N2—H2A	126 (2)	C19—C14—C15	121.6 (2)
C13—N3—C14	108.8 (2)	N3—C14—C15	132.2 (2)
C13—N3—H3A	124.4 (18)	C16—C15—C14	116.0 (3)
C14—N3—H3A	126.6 (18)	C16—C15—H15	122.0
C13—N4—C19	108.6 (2)	C14—C15—H15	122.0
C13—N4—H4A	124.5 (17)	C15—C16—C17	122.3 (3)
C19—N4—H4A	126.9 (17)	C15—C16—H16	118.9
H7A—O7—H7B	103.0 (15)	C17—C16—H16	118.9
H8A—O8—H8B	105.6 (15)	C18—C17—C16	121.9 (3)
H9A—O9—H9B	100.1 (14)	C18—C17—H17	119.1
N2—C1—N1	110.2 (3)	C16—C17—H17	119.1
N2—C1—H1	124.9	C17—C18—C19	116.6 (3)
N1—C1—H1	124.9	C17—C18—H18	121.7
C3—C2—N1	132.0 (2)	C19—C18—H18	121.7
C3—C2—C7	121.6 (2)	C14—C19—N4	106.3 (2)
N1—C2—C7	106.4 (2)	C14—C19—C18	121.6 (2)
C4—C3—C2	116.5 (3)	N4—C19—C18	132.1 (2)
C4—C3—H3	121.8	C24^ii^—C20—C21	120.5 (2)
C2—C3—H3	121.8	C24^ii^—C20—H20	119.7
C3—C4—C5	121.9 (3)	C21—C20—H20	119.7
C3—C4—H4	119.1	C22—C21—C20	120.4 (2)
C5—C4—H4	119.1	C22—C21—H21	119.8
C6—C5—C4	122.0 (3)	C20—C21—H21	119.8
C6—C5—H5	119.0	C21—C22—C23	120.6 (2)
C4—C5—H5	119.0	C21—C22—S2	117.66 (17)
C5—C6—C7	116.4 (3)	C23—C22—S2	121.65 (16)
C5—C6—H6	121.8	C24—C23—C23^ii^	118.9 (2)
C7—C6—H6	121.8	C24—C23—C22	122.86 (19)
N2—C7—C6	132.7 (2)	C23^ii^—C23—C22	118.2 (2)
N2—C7—C2	105.7 (2)	C20^ii^—C24—C23	121.3 (2)
C6—C7—C2	121.6 (3)	C20^ii^—C24—H24	119.3
C12^i^—C8—C9	120.6 (2)	C23—C24—H24	119.3
C12^i^—C8—H8	119.7		

Symmetry codes: (i) −*x*+1, −*y*+1, −*z*+1; (ii) −*x*+1, −*y*+1, −*z*.

### Hydrogen-bond geometry (Å, °)

**Table:**

*D*—H···*A*	*D*—H	H···*A*	*D*···*A*	*D*—H···*A*
N1—H1A···O7^iii^	0.898 (10)	1.820 (12)	2.708 (3)	170 (3)
N2—H2A···O4^iv^	0.887 (18)	1.885 (19)	2.762 (3)	170 (3)
N3—H3A···O3^v^	0.895 (10)	1.937 (12)	2.812 (3)	166 (2)
N4—H4A···O8^i^	0.899 (10)	1.883 (11)	2.771 (3)	169 (2)
O7—H7B···O5^vi^	0.852 (10)	2.169 (19)	2.839 (3)	135 (2)
O8—H8A···O2^vii^	0.844 (10)	1.982 (10)	2.826 (3)	178 (3)
O7—H7A···O1	0.853 (10)	1.999 (12)	2.823 (3)	162 (3)
O8—H8B···O6	0.847 (10)	1.963 (10)	2.809 (3)	176 (3)
O9—H9B···O1^i^	0.863 (10)	2.157 (18)	2.970 (3)	157 (3)

Symmetry codes: (iii) *x*, *y*−1, *z*+1; (iv) *x*+1, *y*, *z*+1; (v) *x*−1, *y*, *z*; (i) −*x*+1, −*y*+1, −*z*+1; (vi) *x*, *y*+1, *z*; (vii) *x*, *y*−1, *z*.
